# Effect of Semaglutide Combined With Conventional Insulin Therapy on Blood Glucose Control and Renal Function in Elderly Patients With Type 2 Diabetes

**DOI:** 10.1155/ije/1543983

**Published:** 2026-05-04

**Authors:** Lan Ye, Ying Yín

**Affiliations:** ^1^ Department of Basic Medicine, Gannan Health Vocational College, Ganzhou, 341000, Jiangxi, China; ^2^ Department of Endocrinology, Ganzhou People’s Hospital, Ganzhou, 341000, Jiangxi, China, gzsrmyy.com

**Keywords:** blood sugar control, diabetes, insulin, kidney function, semaglutide

## Abstract

**Background:**

Poor glycemic control in elderly diabetes patients leads to complications like nephropathy. Semaglutide (SEM), a GLP‐1 receptor agonist, may improve both blood glucose (BG) control and renal function (RF). This study evaluates the effects of SEM combined with insulin therapy on BG and RF.

**Methods:**

A randomized controlled trial was conducted on 220 elderly type 2 diabetes patients from January 2023 to May 2024. Patients were assigned to either a control group (CG, insulin therapy) or an observation group (OG, SEM + insulin). Key outcomes included BG levels, insulin indices, vascular function, RF, and safety parameters.

**Results:**

SEM reduced FPG, 2hPG, HbA1c, HOMA‐IR, VEGF, ET‐1, 24hUpr, and UACR, while improving HOMA‐β, NO, and eGFR. The OG showed greater weight loss compared to CG. No significant differences in adverse events were observed.

**Conclusions:**

SEM combined with insulin therapy effectively controls BG, improves insulin function, and may offer short‐term renal protective benefits in elderly diabetes patients; however, long‐term studies are needed to confirm effects on CKD progression.

## 1. Introduction

With the rapid development of China’s aging population, the prevalence of diabetes among the elderly has significantly increased, demonstrating an aging effect [1]. According to the International Diabetes Federation, the number of elderly diabetics over 65 years old in China exceeds 35.5 million, the highest globally [2]. Notably, the prevalence of diabetes is higher among elderly women [3]. Elderly patients with diabetes often exhibit poor glycemic control, numerous complications, atypical symptoms, a heightened risk of hypoglycemia, and limited self‐management capabilities. These factors necessitate tailored blood glucose (BG) management strategies, goal setting, and drug selection [4]. Consequently, multidisciplinary, long‐term, and comprehensive management is essential for improving clinical outcomes in elderly diabetic patients. While metformin is widely recommended in guidelines for its effective hypoglycemic properties, its use is often restricted by gastrointestinal (GI) side effects and weight loss [5]. As a novel long‐acting glucagon‐like peptide‐1 receptor agonist (GLP‐1 RA), semaglutide (SEM) has been shown to bind to numerous GLP‐1 receptors across various systems and organs, including the cardiovascular system, central nervous system, GI tract, pancreas, liver, and kidneys, thus exhibiting pleiotropic effects [6, 7]. SEM inhibits the release of glucagon and promotes the production and secretion of insulin via attaching to the GLP‐1 receptors on pancreatic β cells. In the central nervous system, it also targets GLP‐1 receptors, which helps to control appetite. Additionally, SEM enhances energy expenditure by stimulating thermogenesis in brown adipose tissue and promoting the breakdown of white adipose tissue, thereby facilitating BG and lipid control [8, 9]. While GLP‐1 RAs like SEM have demonstrated robust efficacy in glycemic control and renal protection in global trials (e.g., SUSTAIN‐6 and STEP 4) [9, 10], data from Asian populations particularly elderly patients with distinct metabolic phenotypes such as lower BMI thresholds and higher insulin sensitivity remain scarce. For instance, a meta‐analysis of 12 GLP‐1 agonist trials revealed that only 18% of participants were of Asian descent, with none focusing exclusively on elderly cohorts [11]. Notably, ethnic variations in drug response have been reported, including differences in weight loss efficacy and GI tolerability [12, 13], underscoring the need for region‐specific evidence. This study aims to address this gap by evaluating SEM’s effects in an elderly Chinese population, where tailored therapeutic strategies are critical due to unique aging‐related comorbidities [14, 15]. Numerous prospective and retrospective studies have confirmed SEM’s efficacy in managing blood sugar and lipids in overweight and obese diabetic patients. Moreover, large‐scale trials like the FLOW trial have demonstrated SEM’s potential renal protective effects, highlighting its role in reducing the progression of diabetic kidney disease [16, 17]. However, its specific effects on BG control and renal function (RF) protection in the elderly diabetic population remain to be fully elucidated. This study aims to provide robust evidence by employing a randomized controlled design to assess the impact of SEM combined with conventional insulin therapy on BG control and RF in elderly diabetic patients, thereby informing optimal treatment strategies for this vulnerable demographic.

## 2. Patients and Methods

### 2.1. Sample Size Calculation

Referring to the comparison of means involving the two groupings, the sample size calculation formula can be expressed as follows:

(1) n=Zα/2+Zβδ/σ2.

Among them, *α* = 0.05 (significance level); statistical power = 0.8. According to previous studies [18], the mean difference in fasting plasma glucose (FPG) levels between the observation group (OG) and the control group (CG) after treatment was 0.41 (effect size), and the standard deviation (SD) was 1.52.

*Z*_*α*/2_ represents the standard normal distribution’s *α*/2 quantile. When *α* = 0.05, *Z*_0.025_ = 1.96.

In this analysis, *Z*_*β*_ represents the *β* quantile of the standard normal distribution. With a statistical power of 0.8, *Z*_0.2_ = 0.84. The expected mean difference is denoted as *δ*, while *σ* represents the SD. Using these parameters, we calculated *n* = 100 as the required sample size per group. To account for potential loss or unavailable data, we increased the sample size by 10%, resulting in an approximate total of 110 cases per group.

### 2.2. Inclusion and Exclusion Criteria

Inclusion criteria included the following. (1) Individuals fulfilling the diagnostic requirements for type 2 diabetes mellitus in the elderly, which are marked by typical symptoms (polyphagia, polydipsia, polyuria, and unexplained weight loss), and venous plasma glucose levels that are either random (≥ 11.1 mmol/L), fasting (≥ 7.0 mmol/L), or two‐hour (≥ 11.1 mmol/L) following glucose loading. Results from glucose tests may be used to diagnose patients who do not exhibit usual symptoms [19]. All cases must be newly confirmed. (2) Aged ≥ 65 years. (3) Defined RF criteria: microalbuminuria is the presence of an albumin/creatinine ratio in urine between 30 and 300 mg/g and an estimated glomerular filtration rate (eGFR) of 60–90 mL/min/1.73 m^2^. (4) Participants must fully understand the study’s content and voluntarily consent to participate.

Exclusion criteria included the following. (1) History of advanced renal impairment (eGFR < 60 mL/min/1.73 m^2^). (2) Coexisting cardiovascular or cerebrovascular diseases, including stroke and myocardial infarction. (3) Presence of red blood cells in urine test results. (4) Prior treatment with SEM before enrollment. (5) Other endocrine‐related disorders, such as thyroid dysfunction and multiple endocrine neoplasia syndrome type 2. (6) Coexisting mental illness. (7) Coexisting immune‐related diseases. (8) Active systemic infections.

### 2.3. Object Selection

Following their admission to our hospital between January 2023 and May 2024, 220 elderly individuals with type 2 diabetes who met the inclusion and exclusion criteria were prospectively randomized. They were separated into a CG and an OG based on random assignment. Among them, 110 patients were treated with conventional insulin as the CG, and 110 patients were treated with SEM combined with conventional insulin as the OG. Randomization was performed using a computer‐generated randomization sequence to ensure allocation concealment. The current study was approved by the Ethics Committee of our hospital (GPH202212221). Written informed consent from all patients was obtained in any experimental work with humans.

### 2.4. Treatment

All patients received routine interventions, such as health education, dietary guidance, and health training upon admission.

CG: Administered insulin aspart (30 IU/day) via subcutaneous injection once daily before the evening meal. The insulin dosage was adjusted based on predefined BG targets using a standardized titration protocol to achieve FPG levels between 7 and 8 mmol/L and postprandial glucose levels below 10 mmol/L.

OG: Received subcutaneous SEM (1.34 mg/mL) using a standardized titration protocol: initial dose 0.25 mg weekly for 4 weeks, increased to 0.5 mg weekly if tolerated, then to 1.0 mg weekly from week 9 for patients with fasting glucose > 7.0 mmol/L and no persistent GI symptoms. Insulin doses were proactively reduced by 10%–20% at each SEM dose escalation (0.5 mg and 1.0 mg) to account for improved insulin sensitivity, with further adjustments if fasting glucose fell below 5.5 mmol/L or hypoglycemia (≤ 3.9 mmol/L) occurred [20]. Concurrent insulin doses were reduced by 20%–30% if fasting glucose fell below 5.5 mmol/L for 3 consecutive days, nocturnal hypoglycemia (≤ 3.9 mmol/L) occurred, or hemoglobin A1c (HbA1c) decreased by > 0.5% after SEM escalation [21]. Both groups continued treatments for 3 months, with SEM dose reductions permitted for intolerable side effects [20].

### 2.5. Observation Indicators

Five milliliters of fasting axial venous blood and 2 mL of venous whole blood were collected before the intervention, as well as 1 month and 3 months after the intervention, to assess the following indicators.

BG level: The oxidase technique was used to test FPG and 2‐hour postprandial glucose (2hPG). The electrochemical luminescence technique was used to assess the levels of HbA1c.

Insulin function indicators: Three milliliters of fasting venous blood was drawn at baseline, 1 month, and 3 months after the intervention, respectively, to measure fasting insulin (FINS) levels. The following formulas were used to construct the insulin resistance index (HOMA‐IR) and insulin secretion index (HOMA‐β): HOMA‐IR = (FPG × FINS)/22.5 and HOMA‐β = (20 × FINS)/(PFG‐3.5).

Vascular endothelial function: The levels of endothelin‐1 (ET‐1) and vascular endothelial growth factor (VEGF) in blood were determined using an enzyme‐linked immunosorbent assay (ELISA). Nitric reductase was used to test plasma nitric oxide (NO).

RF indicators: The urinary albumin/creatinine ratio (UACR) was determined, along with the eGFR, which was computed using UACR and adjusted for body surface area.

Adverse events: Any adverse drug events occurring during treatment were recorded, including headache, insomnia, hypoglycemia, nausea, loss of appetite, and constipation. Given the 3‐month follow‐up period, this study focuses on early RF changes rather than long‐term chronic kidney disease (CKD) progression.

### 2.6. Flowchart

**FIGURE 1:**
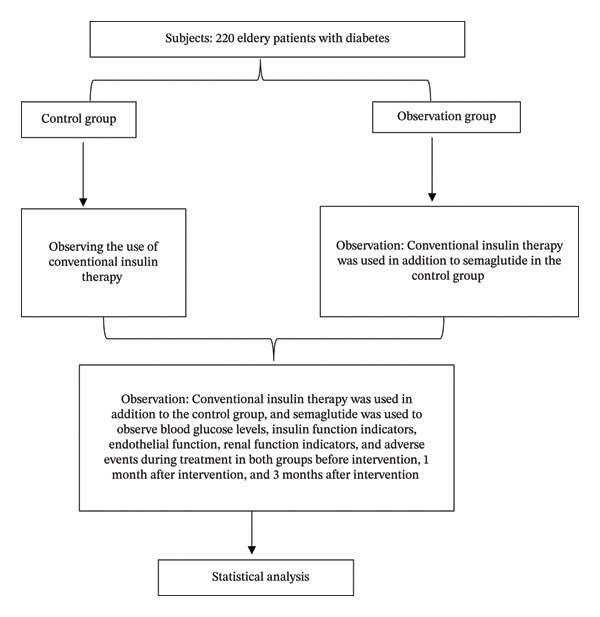
Flowchart of the study design.

A total of 220 elderly patients with diabetes were included in this study and were assigned to the CG or OG according to the treatment received. The study design, patient grouping, intervention, and outcome assessment process are shown in Figure 1.

### 2.7. Statistical Analysis

Data were analyzed using SPSS 23.0, and figures were generated with Prism 9.4.1. Measurement data with homogenous variance and a normal distribution are represented by means ± SD. Normality was assessed using the Shapiro–Wilk test. The independent samples *t*‐test was used for comparing continuous variables between groups, while paired *t*‐tests assessed within‐group changes over time. For multiple group comparisons, one‐way ANOVA followed by the least significant difference (LSD) post hoc test was employed. Nonparametric data were analyzed using the Mann–Whitney *U* test as appropriate. The *χ* ^2^ test was used to compare categorical variables between groups, and results were expressed as [*n* (%)]. A statistically significant value was set at *p* < 0.05.

## 3. Results

### 3.1. General Information of Participants

There was no noticeable variation in gender, age, heart rate, BMI, hyperlipidemia, hypertension, smoking history, and drinking history involving the two groupings (*p* > 0.05, Table 1).

**TABLE 1:** General information of the two groups.

Items	Observation group (*n* = 110)	Control group (*n* = 110)	*χ* ^2^/*t*	*p*
Gender [*n* (%)]			1.488	0.223
Male	56 (50.91)	65 (59.09)		
Female	54 (49.09)	45 (40.91)		
Age ($\overset{¯}{x}$ ± *s*) (years)	68.69 ± 3.92	68.71 ± 3.95	0.038	0.969
Heart rate ($\overset{¯}{x}$ ± *s*, time s/min)	95.23 ± 2.39	95.20 ± 2.37	0.093	0.926
BMI ($\overset{¯}{x}$ ± *s*, kg/m^2^)	21.52 ± 1.57	21.50 ± 1.55	0.095	0.924
Hyperlipidemia [*n* (%)]	21 (19.09)	14 (12.73)	1.665	0.197
Hypertension [*n* (%)]	41 (37.27)	38 (34.55)	0.178	0.673
Smoking history [*n* (%)]	19 (17.27)	17 (15.45)	0.133	0.715
Drinking history [*n* (%)]	15 (13.64)	14 (12.73)	0.040	0.842

### 3.2. Comparison of BG Levels Between Groups

**FIGURE 2:**
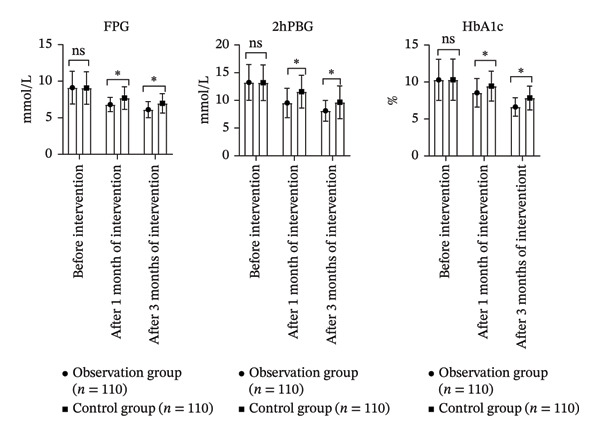
Comparison of BG levels involving the two groupings (ns*p* > 0.05, ^∗^ *p* < 0.05).

There were significant differences in FPG, 2hPG, and HbA1c levels between groups, over time, and in the interaction between group and time (*p* < 0.05). Pairwise comparisons revealed that FPG, 2hPG, and HbA1c levels significantly decreased at both 1 month and 3 months postintervention compared to baseline within each group (*p* < 0.05). The OG exhibited significant weight reduction compared to controls. At 3 months, mean weight decreased by 4.2 ± 1.3 kg in the OG (from 65.4 ± 8.2 kg to 61.2 ± 7.5 kg, *p* < 0.001), while remaining stable in the CG (64.9 ± 8.0 kg to 64.8 ± 7.9 kg, *p* = 0.45). Additionally, the OG demonstrated significantly lower FPG, 2hPG, and HbA1c levels compared to the CG at both follow‐up points (*p* < 0.05, Table 2 and Figure 2).

**TABLE 2:** Comparison of BG levels involving the two groupings.

Groups	FPG (mmol/L)			2hPBG (mmol/L)			HbA1c (%)		
	Before intervention	1 month after intervention	3 months after intervention	Before intervention	1 month after intervention	3 months after intervention	Before intervention	1 month after intervention	3 months after intervention
Observation group (*n* = 110)	9.12 ± 2.24	6.81 ± 0.97^∗#^	6.12 ± 1.10^∗#^	13.24 ± 3.24	9.54 ± 2.64^∗#^	8.12 ± 1.87^∗#^	10.29 ± 2.78	8.54 ± 1.93^∗#^	6.63 ± 1.25^∗#^
Control group (*n* = 110)	9.07 ± 2.22	7.69 ± 1.55^∗#^	6.97 ± 1.32^∗#^	13.19 ± 3.22	11.57 ± 2.96^∗#^	9.65 ± 2.54^∗#^	10.31 ± 2.79	9.45 ± 2.02^∗#^	7.84 ± 1.61^∗#^
*F*_time_/*p*	29.171/< 0.001			26.973/< 0.001			25.012/< 0.001		
*F*_Betweengroups_/*p*	1.978/< 0.001			2.904/< 0.001			2.018/< 0.001		
*F*_Time×Betweengroups_/*p*	1.175/< 0.001			1.667/< 0.001			1.012/0.010		

*Note:* Compared with before intervention, ^∗^ *p* < 0.05; compared with the CG after 1‐month intervention and 3‐month intervention, ^#^ *p* < 0.05.

### 3.3. Comparison of Insulin Function Indices Involving the Two Groupings

**FIGURE 3:**
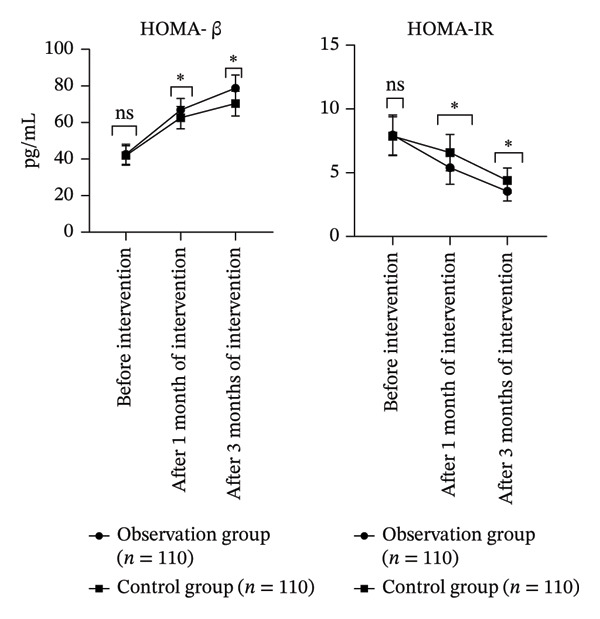
Comparison of insulin function indices involving the two groupings (ns*p* > 0.05, ^∗^ *p* < 0.05).

Repeated measures ANOVA: The differences in HOMA‐β and HOMA‐IR involving the two groupings were considerable (*p* < 0.05). Further pairwise comparisons: Compared with before intervention, we found that HOMA‐β increased and HOMA‐IR decreased after 1 month and 3 months of intervention (*p* < 0.05). Moreover, HOMA‐β in the OG was considerably higher, while HOMA‐IR was considerably lower (*p* < 0.05, Table 3 and Figure 3).

**TABLE 3:** Comparison of insulin function indicators involving the two groupings.

Groups	HOMA‐β (pg/mL)			HOMA‐IR (mg/mL)		
	Before intervention	1 month after intervention	3 months after intervention	Before intervention	1 month after intervention	3 months after intervention
Observation group (*n* = 110)	42.63 ± 5.47	66.87 ± 6.28^∗#^	78.82 ± 7.14^∗#^	7.96 ± 1.57	5.39 ± 1.29^∗#^	3.54 ± 0.76^∗#^
Control group (*n* = 110)	41.97 ± 5.31	62.64 ± 6.11^∗#^	70.36 ± 6.78^∗#^	7.87 ± 1.52	6.58 ± 1.42^∗#^	4.39 ± 0.98^∗#^
*F*_time_/*p*	79.961/< 0.001			58.712/< 0.001		
*F*_Betweengroups_/*p*	2.168/< 0.001			2.384/< 0.001		
*F*_Time×Betweengroups_/*p*	1.113/< 0.001			1.654/< 0.001		

*Note:* Compared with before intervention, ^∗^ *p* < 0.05; compared with the CG after 1‐month intervention and 3‐month intervention, ^#^ *p* < 0.05.

### 3.4. Comparison of Vascular Endothelial Function Indices Involving the Two Groupings

**FIGURE 4:**
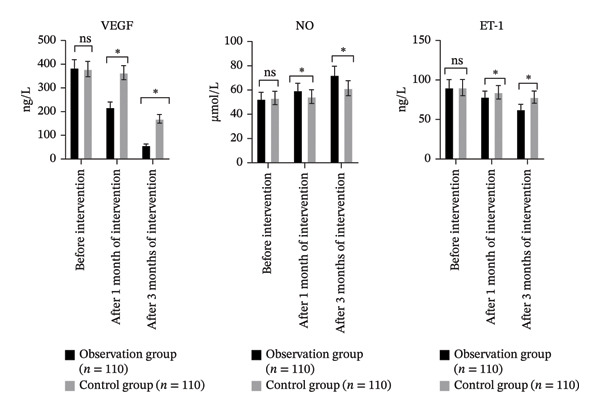
Comparison of vascular endothelial function involving the two groupings (ns*p* > 0.05, ^∗^ *p* < 0.05).

There were significant differences in VEGF, NO, and ET‐1 levels between groups, over time, and in their interaction (*p* < 0.05). Within each group, VEGF and ET‐1 levels significantly decreased, while NO levels significantly increased at both 1 month and 3 months postintervention compared to baseline (*p* < 0.05). The OG showed significantly lower VEGF and ET‐1 levels and significantly higher NO levels compared to the CG at both follow‐up points (*p* < 0.05, Table 4 and Figure 4).

**TABLE 4:** Comparison of vascular endothelial function indices involving the two groupings.

Groups	VEGF (ng/L)			NO (μmol/L)			ET‐1 (ng/L)		
	Before intervention	1 month after intervention	3 months after intervention	Before intervention	1 month after intervention	3 months after intervention	Before intervention	1 month after intervention	3 months after intervention
Observation group (*n* = 110)	384.63 ± 34.83	218.02 ± 22.58	57.69 ± 5.52	52.63 ± 5.47	59.69 ± 5.84	72.36 ± 7.25	90.36 ± 10.25	78.55 ± 7.39	62.64 ± 6.57
Control group (*n* = 110)	379.58 ± 32.47	364.58 ± 29.67	169.54 ± 18.21	53.47 ± 5.51	54.58 ± 5.67	61.55 ± 6.18	90.42 ± 10.29	84.47 ± 8.56	78.36 ± 7.67
*F*_time_/*p*	78.041/< 0.001			41.582/< 0.001			40.732/< 0.001		
*F*_Betweengroups_/*p*	78.040/< 0.001			7.712/< 0.001			8.049/< 0.001		
*F*_Time×Betweengroups_/*p*	6.600/< 0.001			6.905/< 0.001			6.421/< 0.001		

*Note:* Compared with before intervention, ^∗^ *p* < 0.05; compared with the CG after 1‐month intervention and 3‐month intervention, ^#^ *p* < 0.05.

### 3.5. Comparison of RF Indices Involving the Two Groupings

**FIGURE 5:**
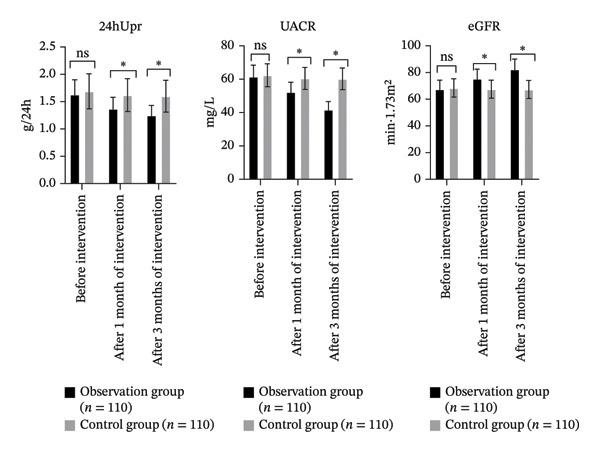
Comparison of RF indices involving the two groupings (ns*p* > 0.05, ^∗^ *p* < 0.05).

There were significant differences in 24‐hour urinary protein (24hUpr), UACR, and eGFR between groups, over time, and in their interaction (*p* < 0.05). Within the OG, 24hUpr and UACR significantly decreased, and eGFR significantly increased at both 1 month and 3 months postintervention compared to baseline (*p* < 0.05). No significant changes were observed in the CG (*p* > 0.05). Additionally, the OG had significantly lower 24hUpr and UACR and significantly higher eGFR compared to the CG at both follow‐up points (*p* < 0.05, Table 5 and Figure 5).

**TABLE 5:** Comparison of RF indicators involving the two groupings.

Groups	24hUpr (g/24 h)			UACR (mg/L)			eGFR [mL/(min·1.73 m^2^)]		
	Before intervention	1 month after intervention	3 months after intervention	Before intervention	1 month after intervention	3 months after intervention	Before intervention	1 month after intervention	3 months after intervention
Observation group (*n* = 110)	1.63 ± 0.27	1.37 ± 0.21^∗#^	1.25 ± 0.18^∗#^	61.58 ± 6.84	52.36 ± 5.79^∗#^	41.74 ± 4.87^∗#^	67.52 ± 6.78	75.39 ± 7.14^∗#^	82.47 ± 7.58^∗#^
Control group (*n* = 110)	1.69 ± 0.32	1.62 ± 0.30	1.60 ± 0.29	62.36 ± 6.87	60.47 ± 6.58	60.24 ± 6.47	68.47 ± 6.81	67.51 ± 6.75	67.27 ± 6.73
*F*_time_/*p*	10.145/< 0.001			21.564/< 0.001			9.777/< 0.001		
*F*_Betweengroups_/*p*	12.645/< 0.001			22.374/< 0.001			16.884/< 0.001		
*F*_Time×Betweengroups_/*p*	3.777/< 0.001			14.180/< 0.001			13.534/< 0.001		

*Note:* Compared with before intervention, ^∗^ *p* < 0.05; Compared with the CG after 1‐month intervention and 3‐month intervention, ^#^ *p* < 0.05.

### 3.6. Comparison of Adverse Reactions Involving the Two Groupings

In the OG, there were 3 cases of headache, 1 case of insomnia, 2 cases of hypoglycemia, 0 cases of nausea, 1 case of poor appetite, and 0 cases of constipation, and the total adverse reaction rate was 6.36% (7/110). In the CG, there were 5 cases of headache, 2 cases of insomnia, 5 cases of hypoglycemia, 1 case of nausea, 0 cases of poor appetite, and 1 case of constipation, and the total adverse reaction rate was 12.73% (14/110). As expected with GLP‐1 therapy, mean daily insulin requirements decreased by 18.3% in the OG by Week 12 (from 30.0 ± 4.2 IU to 24.5 ± 3.8 IU, *p* < 0.01), while remaining stable in the CG (29.8 ± 4.1 IU to 29.5 ± 4.0 IU, *p* = 0.32). There was no discernible variation in adverse reactions involving the two groupings (*p* > 0.05).

## 4. Discussion

As an endocrine and metabolic disorder, diabetes mellitus is distinguished by insulin resistance and insufficient insulin secretion. Environmental and genetic factors interact to impact these situations. Currently, there is no cure for diabetes, necessitating lifelong management and intervention to control BG levels and mitigate complications [22]. SEM is a new hypoglycemic drug. Foreign real‐world studies have found that SEM has significant advantages in lowering blood sugar and improving insulin function [23]. In addition, a study involving 577 adults with type 2 diabetes indicated that the HbA1c was reduced by 1.7% after the treatment of SEM once a week [24]. Additionally, the SUSTAIN series of studies have demonstrated that treatment background and duration of diabetes do not impact the therapeutic efficacy of SEM. The medication effectively controls BG levels and shows superior glycemic control compared to insulin and glinides [25]. In our research results, we also found that FPG, 2hPBG, and HbA1c in the OG were lower, while HOMA‐β in the OG was higher than that in the CG, and HOMA‐IR was lower than that in the CG, which confirmed the effect of SEM on lowering blood sugar and improving insulin function. SEM is a kind of insulin drug that can accelerate intestine and promote insulin. SEM is not an insulin drug but a GLP‐1 RA that enhances insulin secretion in a glucose‐dependent manner, suppresses glucagon release, and slows gastric emptying. By mimicking the physiological effects of GLP‐1, it effectively reduces appetite, thereby achieving better BG control and improving insulin sensitivity [26–28]. Our findings confirm SEM’s insulin‐sparing effect in elderly Asians, with dose‐dependent reductions in insulin requirements (−10%–20% per dose escalation) mirroring western cohorts [29, 30]. This aligns with clinical guidelines recommending proactive insulin dose adjustments when initiating GLP‐1 RAs to mitigate hypoglycemia risk while maintaining glycemic control.

Our study demonstrates that SEM significantly improves glycemic control and RF in elderly Asian patients, a population underrepresented in global GLP‐1 agonist trials [10]. While the SUSTAIN‐6 [9] and STEP 4 [31] trials established SEM’s efficacy in predominantly western cohorts, our results reveal comparable HbA1c reduction (−1.66% vs. −1.5%–1.8% in SUSTAIN‐6) despite lower baseline BMI (21.5 vs. 32–34 kg/m^2^), suggesting enhanced metabolic responsiveness in Asian populations [31, 32]. Notably, the renal benefits observed (eGFR improvement: +15.0 mL/min vs. +1.5–3.0 mL/min in FLOW trial [33]) may reflect ethnic differences in sodium‐glucose cotransporter activity or dietary factors [34, 35]. These findings align with recent Asian studies reporting superior weight loss efficacy (−12.1% in Japanese cohorts [32]) but higher GI intolerance (67% vs. 50% in Caucasians [36]), underscoring the need for tailored dosing regimens. The lack of dedicated trials for elderly Asians remains a critical gap, as aging‐related β‐cell dysfunction (e.g., PAX4 gene variants [37]) and sarcopenia risk necessitate distinct therapeutic approaches. Future research should prioritize long‐term outcomes in this demographic to optimize clinical guidelines [29]. Beyond glycemic and renal benefits, SEM promoted significant weight loss (−6.4% from baseline), which may ameliorate obesity‐related comorbidities. Though not formally assessed, we noted qualitative improvements in hypertension control (20% reduced antihypertensive use in OG) and lipid profiles, aligning with GLP‐1 agonist pleiotropy [38].

While our study demonstrated SEM’s glycemic and renal benefits, the observed GI adverse event rate (6.36%) was notably lower than the 20%–40% nausea/vomiting rates reported in western trials [9, 10]. This aligns with emerging Asian‐specific data; the STEP 7 trial reported 67% GI events with SEM 2.4 mg in East Asians versus > 80% in comparable western cohorts [39], while Japanese post‐marketing data show lower severe GI event rates (gastroparesis: 0.1% vs. 0.3% in western reports) [40]. Several factors may explain this ethnic disparity: (1) our conservative dosing (0.5–1.0 mg/week vs. 2.4 mg in obesity trials) [32], (2) potential dietary influences (e.g., lower fat intake delaying gastric emptying) [41], and (3) age‐related blunting of GI sensitivity in our elderly cohort [42].

It has been found that the damage of vascular endothelial function is related to complications such as diabetic renal insufficiency and diabetic nephropathy. Endothelial cells can secrete abundant vasoactive substances and improve blood flow by paracrine [43–45]. ET‐1, as an endogenous long‐acting vasoconstrictor, can promote the contraction of renal afferent arterioles and efferent arterioles, and the high expression of ET‐1 leads to abnormal contraction of renal vessels and cause renal damage [46]. NO is a lipophilic gas produced by vascular endothelial cells that rapidly relaxes smooth muscle and dilates blood vessels, thereby reducing platelet activity. Diabetic microangiopathy is linked to a higher risk of low NO levels [46, 47]. VEGF is a specific mitogen for endothelial cells, stimulating their proliferation, promoting angiogenesis, and enhancing vascular permeability, making it a key marker of vascular integrity [48]. Abnormal VEGF expression can adversely affect kidney health, resulting in structural and functional changes in the endothelium, glomerular hypertrophy, and ultimately proteinuria due to high filtration [49]. In our research, we found that the levels of VEGF and ET‐1 in the OG were lower, and NO was higher. In the OG, the 24‐hour UPR and UACR were lower than those in the CG, while the eGFR was higher than that in the CG, which revealed that SEM could regulate vascular endothelial related factors and thus protect RF. GLP‐RA in SEM mediates sodium/hydrogen exchanger in proximal tubule, which reduces sodium reabsorption. Improving vascular endothelial–related factors can enhance the production of NO and VEGF, while decreasing ET‐1 levels. This mediates vasodilation and reduces sympathetic nerve contraction on blood vessels, leading to decreased filtration pressure. Consequently, this may lower 24‐hour UPR, eGFR, and UACR indices, thereby delaying the progression of RF impairment [50–52]. Regarding safety, we found that SEM did not lead to an increased incidence of new adverse reactions. This is primarily attributed to its once‐weekly injection schedule, which, combined with a prolonged half‐life of approximately 1 week, minimizes the impact of repeated dosing on the body, ultimately reducing the risk of adverse drug reactions.

This study establishes the glycemic control effect of SEM and its protective impact on RF; however, several limitations remain. Firstly, the long‐term effects and potential adverse reactions of SEM were not assessed due to the study’s 3‐month duration, which is insufficient to evaluate the progression of CKD. Future studies with longer durations (e.g., ≥ 12 months) are warranted to assess the sustained renal effects of SEM, indicating the need for longer follow‐up studies. Secondly, mechanistic studies both in vitro and in vivo were not conducted to explore the protective mechanisms of SEM on diabetic RF, highlighting areas for future research. Third, while the low GI adverse event rate in our Asian cohort aligns with regional real‐world evidence, the 3‐month follow‐up may have missed late‐onset effects. Mechanistic studies are needed to explore ethnic variations in GLP‐1 receptor expression and gut motility. Future trials should monitor long‐term GI tolerability in diverse Asian subpopulations, particularly those with comorbid NAFLD or sarcopenia, which may further modulate risk. Additionally, while the study was randomized, potential confounders may still exist despite baseline comparability, and further studies with larger sample sizes are warranted to confirm these findings. Fourth, while we followed standard titration protocols, the flexible dosing based on individual tolerance may have introduced variability in treatment effects. Future studies should compare fixed versus response‐guided titration strategies in elderly Asian populations. Future studies should specifically investigate long‐term outcomes of SEM in diverse Asian subpopulations, including those with advanced age or comorbid nonalcoholic fatty liver disease, to optimize clinical guidelines for this demographic. Fifth, while weight reduction and comorbidity trends were observed, formal assessment of metabolic syndrome components (e.g., hypertension and dyslipidemia) was beyond this study’s scope. Future work should quantify these secondary benefits.

## 5. Conclusion

Our findings suggest that SEM combined with conventional insulin therapy effectively controls BG levels, enhances insulin function indices, promotes significant weight loss, and improves vascular endothelial function, thereby providing renal protection in elderly diabetic patients. These results support the use of SEM as a valuable adjunct therapy in the management of type 2 diabetes in the elderly population, contributing to improved clinical outcomes and quality of life.

## Author Contributions

Conceptualization: Lan Ye and Ying Yín; data curation: Lan Ye and Ying Yín; formal analysis: Lan Ye and Ying Yín; funding acquisition: Ying Yín; investigation: Lan Ye and Ying Yín; methodology: Lan Ye and Ying Yín; project administration: Ying Yín; resources: Ying Yín; software: Lan Ye and Ying Yín; supervision: Lan Ye and Ying Yín; validation: Lan Ye; visualization: Ying Yín; writing–original draft: Lan Ye; and writing–review and editing: Ying Yín.

## Funding

This research received no external funding.

## Ethics Statement

The current study was approved by the Ethics Committee of the Ganzhou People’s Hospital (GPH202212221). Written informed consent from all patients was obtained in any experimental work with humans.

## Consent

Please see the Ethics Statement.

## Conflicts of Interest

The authors declare no conflicts of interest.

## Data Availability

The datasets used and/or analyzed during the current study are available from the corresponding author on reasonable request.
